# An Integrated Care Strategy for Pre-schoolers with Suspected Developmental Disorders: The Optimus Co-design Project that has Made it to Regular Care

**DOI:** 10.5334/ijic.5494

**Published:** 2021-04-15

**Authors:** Anna Sarkadi, Anton Dahlberg, Kajsa Leander, Moa Johansson, Johanna Zahlander, Anna Fäldt, Robert S. Kristiansson, Kine Johansen

**Affiliations:** 1 Uppsala University, SE

**Keywords:** preschool children, neurodevelopment disorder, co-design, team care, quality improvement

## Abstract

**Introduction:**

Multiple neurodevelopmental problems affect 7–8% of children and require evaluation by more than one profession, posing a challenge to care systems.

**Description:**

The local problem comprised distressed parents, diagnostic processes averaging 36 months and 28 visits with 42% of children >4 years at referral to adequate services, and no routines for patient involvement. The co-design project was developed through a series of workshops using standard quality improvement methodology, where representatives of all services, as well as parents participated.

The resulting integrated care model comprises a team of professionals who evaluate the child during an average of 5.4 appointments (N = 95), taking 4.8 weeks. Parents were satisfied with the holistic service model and 70% of children were under 4 at referral (p < 0.05). While 75% of children were referred, 25% required further follow-up by the team.

**Discussion:**

The Optimus model has elements of vertical, clinical and service integration. Reasons for success included leadership support, buy-in from the different organisations, careful process management, a team co-ordinator, and insistent user involvement.

**Conclusion:**

Evaluating multiple neurodevelopmental problems in children requires an integrated care approach. The Optimus care model is a relevant showcase for how people-initiated integrated care reforms can make it into usual care.

## Background

Neurodevelopmental disorders, such as Attention Deficit Hyperactivity Disorder (ADHD), Developmental Coordination Disorder (DCD), autism spectrum disorder (ASD), developmental language disorder, conduct disorder, and learning impairment in children are common [1]. The estimated prevalence rate of their early symptoms, leading to evaluation by a professional is about 7–8% [2], with co-occurring symptomatology being a rule rather than the exception [3,4]. Early symptoms of delayed development may signal the presence of an underlying treatable disorder and early detection is therefore crucial to enable timely interventions that can improve outcomes and divert negative pathways [5]. Early detection of disability can also provide an opportunity to establish early parental support [5] which is known to have a lasting positive impact on the parent’s mental health [6].

Children with complex needs comprise a great challenge, not only to themselves and their families, but also to health systems [7], as having difficulties in several domains, requires evaluation and intervention by more than one professional. Although this is well-known, today’s healthcare moves towards being increasingly specialised and catering to the needs of children with one disorder only [1,8]. Service fragmentation and a silo approach is frequently reported to cause difficulties or delays in accessing services [9], and hence, to improve health and systems outcomes as well as to provide high quality care, care coordination is vital [9]. Besides being inefficient and insecure, the current organisation of care is also demanding for the families who have to juggle multiple roles to coordinate and fight for the adequate care of their child [10,11]. Not surprisingly, parents caring for children with developmental difficulties often describe a burdensome life situation with high levels of stress that affect the family’s well-being [1,10,12], and they are asking for integrated approaches that includes them in the care for their child [13].

Fragmented and uncoordinated care has been identified as one of the most wasteful areas of spending of healthcare resources [14]. Due to both the nature of their problems and the nature of the fragmented care provided, children with neurodevelopmental disorders utilize more healthcare services than typically developing children [4,7]. While more care might be a necessity, an integrated health care service for this population, in line with the WHO-definition [15], could lead to considerable reduction in health care spending/waste, along with improvements in the quality, safety, and perception of care provided for families raising children with neurodevelopmental disorders. An integrated care approach, with the aim to address fragmentation in patient services and enable better coordination and more continuous care [16], was therefore deemed as an adequate framework for this quality improvement project.

Although a multiprofessional approach is regarded as necessary when evaluating children with a developmental delay, there is not a single agreed model on how best to do this. An example of successful integration and maintenance of a service to assess developmental delay has been reported from the Isle of Wight [17]. Another example is a team assessment model for children 2–10 years of age from Kerala, India [18]. A scoping review found certain components facilitating sustainability of integrated behavioural healthcare: process-related issues, such as the role of interprofessional communication and implementation support; content-related issues, such as the use of clear protocols; and organizational issues, such as co-employment of the care providers [19].

## The Optimus integrated care model for multidisciplinary assessment

The authors of this paper had the role of embedded researchers [20], with K.J. and A.F. working as clinicians seeing children with multiple developmental problems at the time and A.S. on a clinical quality improvement research grant to lead the process. The three formed the initial project team, before inviting new members, as specified below.

### Problem statement

Swedish healthcare is organised across three sectors of health service (primary, secondary and tertiary) and is publicly financed. The Swedish health care system is regulated by the Health Care Act aiming to achieve good health and equal care for the entire population and prioritizing those in greatest need of healthcare [21].

Almost all children below the age of 5 years attend the child health services (primary care) and when problems are detected, e.g., at the language screening at 2.5 years of age, the child is referred to specialized care (secondary or tertiary care). In the county of Uppsala, specialised care is organized at the Uppsala University Hospital mainly according to professions or specialization such as paediatric neurology, speech and language pathology, psychology, etc, which implies that the child can be referred to different professions at different clinics when concerns are detected in more than one area of development. When there is sufficient suspicion of a neurodevelopmental diagnosis, the child can be referred to the Habilitation services (a service for children with chronic functional impairment requiring specialised services, divided into motor disabilities, intellectual disabilities and autism spectrum disorders) or the Child- and Adolescent Psychiatry services (for ADHD or significant child psychiatric morbidity) for further evaluation and treatment. If a child shows early and severe signs of communication or developmental disorder and there is a strong suspicion of e.g., autism spectrum disorder, the child health services can refer the child for a “fast track” evaluation by a psychologist and on to Habilitation services. Healthcare for children is, regardless of the sector or services, free of charge.

In the Uppsala Region, professionals encountering children with suspected developmental disorder, albeit not enough to cause severe disability or have a clearly defined “label”, expressed increasing frustration that the care they were able to provide was neither equitable nor achieving priority of those with the greatest needs. The professionals felt that families suffered from the fragmentation and lack of coordination of care; parents had described to them the burden of visiting a number of clinics, receiving different and sometimes contradictory advice. Although professionals felt they did not have the power to change the situation in their respective ‘silos’, these parents’ accounts gave the idea to obtain a more systematic description of the situation of children with problems in more than one area of development through a qualitative study and care data -and process analysis.

#### Distressed parents

In 2015, we conducted a qualitative interview study with parents of children (N = 8) who had seen multiple professionals due to developmental problems in their children [22]. The results showed that parents experienced frustration with the uncoordinated and inconsistent care of their children. Parents did not understand the different roles of professionals involved, felt insecure, anxious, and lost in the process, had no one to turn to with questions and that no one seemed to have a plan for their child.

Results from the parent interviews were presented to a high-level board of directors called the Centre for Functional Impairment, the purpose of which was to enhance cross-organisational collaboration in the health care region. The board appointed a group of professionals from the different sectors and disciplines to compile evidence from administrative data to see if there was a rationale to initiate a project.

#### Inefficient diagnostic processes

We conducted a series of case studies in 2016, where each profession chose a child from their caseload, for whom they were primary contact in the recent 12 months, and reviewed all other contacts the child had, using electronic medical records. The case studies mirrored parent’s experiences with children having contact with many clinics and professions without any coordination, inefficient diagnostic processes and no routines for involving parents in the care planning. Professionals also noted that certain “unofficial” case conferencing usually occurred regarding children with complex symptoms, but without proper documentation or time allowance. We then reviewed administrative data for clinician visits for children who had been referred to Habilitation services during 2015 with neurodevelopmental disorders (n = 93). On average, the time between the first and the last visit at the Children’s Hospital before referral (proxy for the evaluation and care planning process) was 36 months (range 2–76 months) with an average of 28 visits to different professionals (4–185 visits).

#### Late referral to adequate services

The age distribution of children 1–6 years of age referred to Habilitation services 2009–2015 with neurodevelopmental disorders (N = 99) showed that only 58% were less than four years of age and 22% were 5–6 years old, i.e., many of them too old for effective early intervention (see ***Figure 2*** for age distribution at baseline).

#### Inefficient referral processes

Of the total referrals to the Habilitation services of children with neurodevelopmental disorders 2009–2015 (N = 120, including < 1- and 6–7-year-olds) 16% were refused either due to missing information in the referral or the child not considered eligible for the services at the time of referral. These kinds of refusals generate waste in the system and cause significant delay in adequate interventions for the children and families involved.

#### No routines for involving parents in care planning

While the Children’s Hospital has an advisory council consisting of parents and children for patients in tertiary care, there was no routine for involving families in the paediatric outpatient services. We therefore, set out to involve parents in all steps of the process, from developing and designing the care model to continuously improving it.

### Development process of the Optimus integrated care model

The results described in the “Problem description” section above were presented to the board, which then decided to authorise a two-year project for developing a care model that would solve the problem for children who need multidisciplinary evaluation for neurodevelopmental disorders.

### Benchmarking

A multidisciplinary model of assessment, the “school function clinic”, was developed at the Royal Children’s Hospital in Melbourne to diagnose behavioural problems/ADHD in children 4–8 years of age. An audit and evaluation of the clinic was published in 2010 [23]. The clinic team included paediatricians, clinical psychologists and a special educator. A triage procedure was employed and only children with sufficient symptoms and a need for a multidisciplinary assessment were accepted. The special educator visited the school prior to the clinic appointment, observing the child. A full assessment was carried out during a single day at the clinic. Verbal feedback was given to the parents following the case conference, and a written report provided to the parents and involved professionals within two weeks. Notably, there was no speech or occupational therapist in the team and 28% and 11% of the children, respectively, required additional assessment by these professionals. The first author (A.S.) visited the Royal Children’s Hospital on multiple occasions to study and observe the team’s work and inquire about their recommendations for further development.

We also contacted the Gillberg Centre in Gothenburg [1] and visited the Child and Adolescent Psychiatry services in Farsta, Stockholm, both being Swedish examples of a multidisciplinary team for children with developmental problems. Just like the school function clinic, neither of these worked across different services – the different professionals were instead all employed in the same service. They also had the same professionals as the Melbourne clinic.

### Developing the model

Paediatricians, as well as other employed staff were compensated by the organisation; the senior sponsor allowed the team 5% of their time to develop the care model during the first year of the project and 5% of their time to see children in the team on an ongoing basis.

The integrated care model itself was developed through a series of workshops, where representatives from all services (including municipal preschools) as well as patient representatives, from the NGO Attention, participated. The process followed best practice in quality improvement, as described by Nelson et al. [24]. The patient representatives contributed greatly to the process by educating professionals on parental priorities, needs, and challenges, given that many of the parents raising children with neurodevelopmental impairment may have cognitive challenges themselves, such as ADHD. Specific details, where patient representatives contributed, included suggesting an initial phone call to families to see if they are psychologically ready for a full evaluation, including the results it might give; advice about the wording and format of the clinic invitation, featuring specific guidance, parking details, etc; the suggestion to support parents with taking notes and giving them a written summary of the evaluation, emphasising *both* their child’s strengths and challenges and a written plan with contact details going forward; and planning visits without children to discuss sensitive matters.

### Description of the Optimus integrated care model

**Figure 1:**
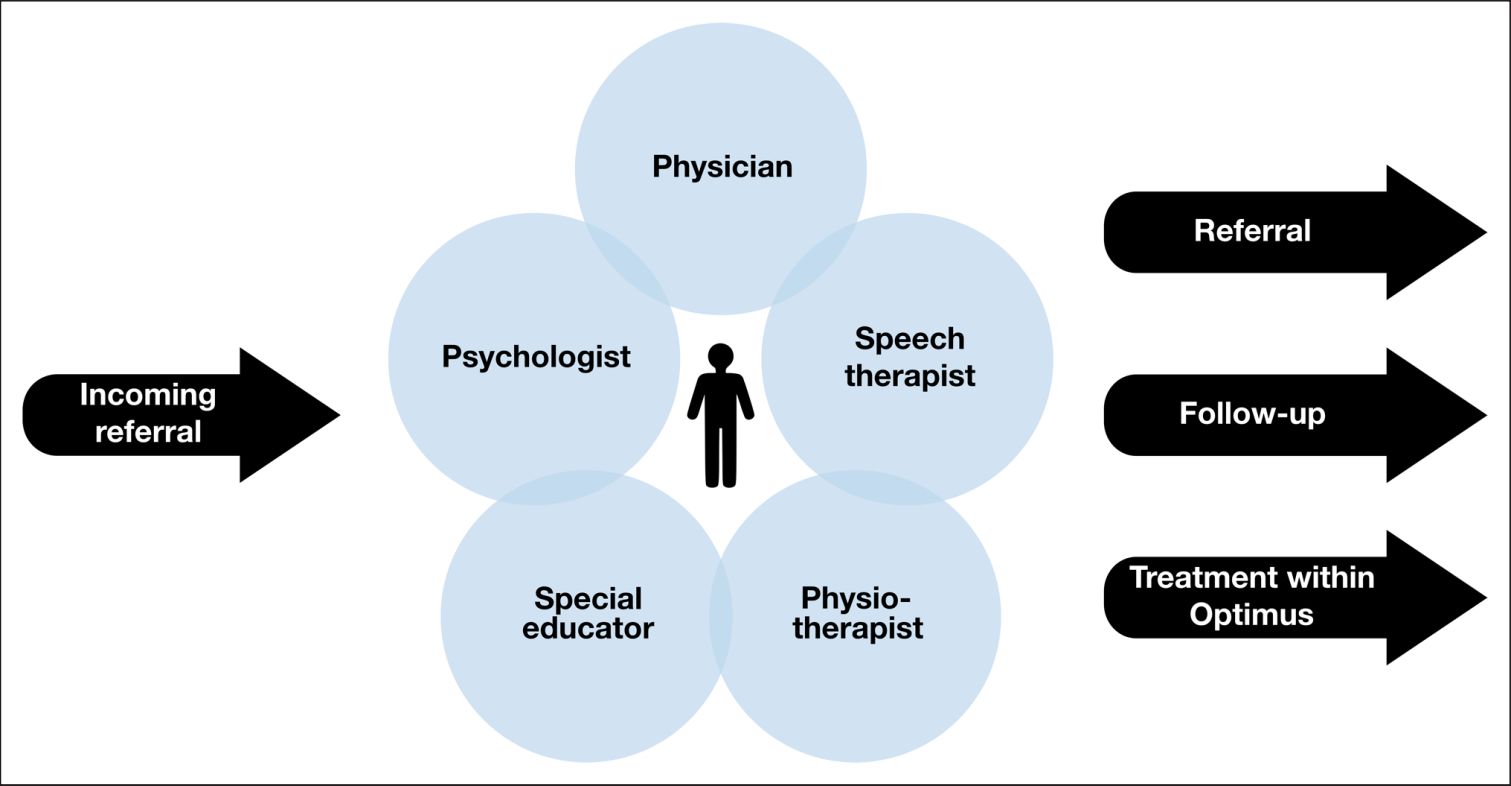
An illustration of the Optimus integrated care model.

The Optimus integrated care model consists of a physiotherapist, a paediatrician, a speech therapist, a number of special educators serving different preschools, and two psychologists (***Figure 1***). One of the professions has 20% of their time to act as a team coordinator. An occupational therapist is connected to the team but is not part of the evaluation process. A delegate from the Habilitation services referral group also partakes in the team conference for discussion. The special educator observes the child in the preschool setting and interviews the child’s preschool teacher prior to attending and reporting to the case conference.

Parents are contacted prior to their visit to ascertain their readiness and availability to participate in the comprehensive assessment. Parents receive all scheduled appointments at once, sent to them per post, including a standardised assessment form [25] that they are asked to fill in and bring along to the clinic.

The members of the team work together half a day per week. Each patient appointment usually takes 30–60 minutes, depending on the professional seen. Visits are scheduled in the following order: anamnesis with a psychologist and paediatrician, without the child present; clinic visits for the child with multiple professions; and a final feedback session, without the child. For the team, the half-day ends with a 75-minute case conference to discuss referrals and ongoing evaluations.

The Optimus integrated care model team was launched in September 2016. Continuous modifications have been made in the team’s routines, but the general concept stayed the same. Modifications included further improving information to parents prior to scheduling their visits, testing (and keeping) common visits to the speech and physiotherapist in the team, development of a common observation scheme for special educators conducting preschool observations, better structure for the case conference, and online or in person participation in the case conference of psychologists or other professionals previously involved in the child’s evaluation. Continuous efforts were made to involve child psychiatry services without success.

### Evaluation

#### Measures

The evaluation measurements were guided by the Institute for Healthcare Improvement framework [26] for measuring quality improvement: *outcome measures, process measures*, and *balancing measures*. Outcome measures consisted of *child age* at referral and *patient (parent) experience*. Process measures included *care process data*, while balancing measures included looking for unintended consequences or undesired effects on other parts of the system. Finally, patient centred care requires effective *user involvement*, and therefore this process was also documented.

#### Data collection

Child age was collected from referrals in the electronic medical record. An online client satisfaction questionnaire was sent to all parents who had their follow-up sessions Nov 2016 – September 2017 (N = 28). Care process data were collected from patient records. Notes and presentations from the project documented the process of user involvement.

Fisher’s exact test was used to compare frequency distribution of age groups referred to Habilitation services before and after the establishment of the team. Process data and clinical data was analysed using descriptive statistics, covering the period from March 2017 to January 2020.

#### Ethical Considerations

According to chapter 5 § 4 in the Swedish Health Care Act, providers of healthcare are obliged to develop the quality of their services. The quality improvement effort described in this article was conducted under the realm of this law, commissioned by the regional leadership. Although evaluation of outcomes and process was planned, the project was not a research project and therefore there was no requirement for a formal ethical approval. Only professionals involved in the care of patients or responsible for referral administration had access to patient records.

## Results from the evaluation of the Optimus integrated care model

### Outcome measures

#### Clinical outcomes

A majority (58%) of the 95 children seen by the team were referred with the probable diagnoses of autism and/or intellectual disability (the final diagnosis of autism is made by the Habilitation services in Uppsala county), whereas 9% were referred to Child and adolescent psychiatry.

**Figure 2:**
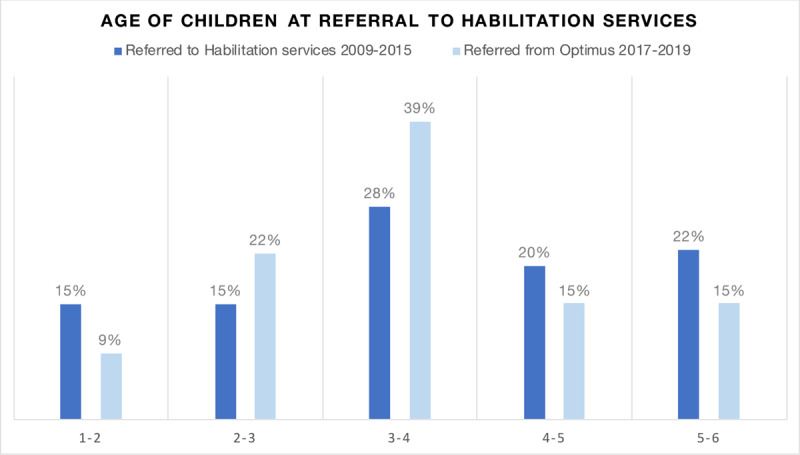
Percentages of children in different age groups at referral to Habilitation services.

After introducing the Optimus integrated care model children were referred to the Habilitation services at an earlier age. Before introducing Optimus (2009–2015), a total of 58% of the children were 48 months or younger when referred, compared to 70% among those referred by Optimus March 2017 – January 2020 (***Figure 2***). Changes were statistically significant, *p* = .026. The mean age for referral after introducing the new work model was 43.6 months. The greatest difference was observed among the proportion of 3–4-year-olds referred, where these constituted 39% of the children compared to 27% previously.

#### Patient experience – questionnaire data

An online client satisfaction questionnaire was sent to 28 parents during the first year. Parent overall satisfaction (N = 20, response rate 71%) was high (3.8 of 4) as well as perceived safety of the diagnostic process (3.8). Parents perceived team roles to be very clear (3.9) and the team’s process as transparent (3.6). Most parents (70%) perceived that there was a clear care plan for their child after the evaluation process. However, only 60% of parents indicated they knew who to contact if they had questions. A majority of the respondents (80%) indicated that they had been worried about their child’s development and 80% that their child already had extra resources, such as an assistant or special educator, in the preschool.

### Process measures

#### Care process data

The average time for the diagnostic process from first to last visit was 4.8 weeks and resource use was 5.4 clinician appointments per child (N = 95). Among the referrals to Habilitation services (N = 51), the refusal rate was 6.5% compared to 16% before the Optimus integrated care model.

**Figure 3:**
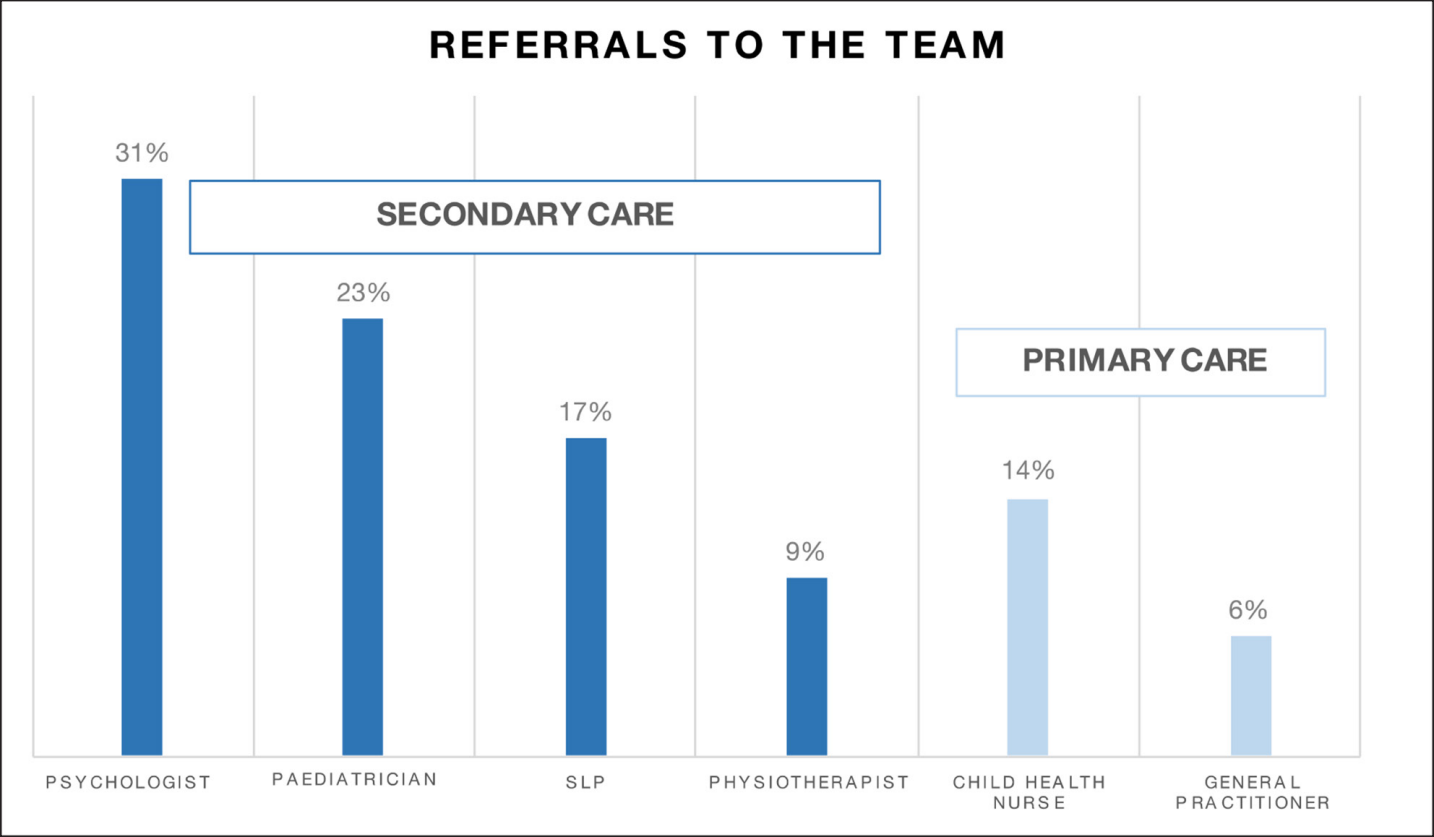
Professionals referring to the Optimus integrated care model.

Referrals came primarily from psychologists and paediatricians (***Figure 3***), i.e., professionals in secondary and/or tertiary care, although 20% came from primary care (GP or child health nurse). Average waiting time from referral to first visit was 2.5 months. The referral influx reached a steady state during the study period, and remained at operative levels, not causing long waiting times.

#### Costs

In addition to the clinic visits, we count 0.25 hours of the case conference, and about 0.50 hours of the coordinator’s time per family, totalling approximately 6 hours and 15 minutes per child. In addition, the special educators spent approximately 2.5 hours on average observing the child at the preschool (including travel) and participating in the case conference, but there were no formal records kept on special educators’ time use. Paediatrician time per child increased compared to the previous routine. In addition, a new cost was introduced due to the coordinator function, comprising 20% working time. To avoid unintended opportunity cost due to crowding out, special care was taken to survey waiting lists of allied health professionals represented in the team and assistance offered to alleviate case load. No societal costs or costs for families were included.

#### Balancing measures

Paediatrician time per patient increased compared to previous routines. Additional time comprised the case conference and the feedback session with the family. However, the case conference was perceived to increase diagnostic quality and contribute to the continuous education of all professionals involved and replaced previous “unofficial” conferencing. Regarding the feedback session, the team decided to involve the paediatrician in sessions only when motivated, such as when difficult messages to parents were to be delivered and care plan-related options and consequences discussed. According to the paediatrician, this reduced the previously frequent number of later appointments by the family for lingering questions and concerns.

Every fourth child was assessed as having complex needs without meeting the referral criteria for specialist services. Neither could their needs be met in primary care. In these cases, the team constructed an individualised care plan utilising available community, preschool and health care resources and with a follow-up appointment with the team six months later. During this time period, the children and parents received tailored interventions from members of the team, including parenting support, speech and language interventions, and physiotherapy, based on their individual needs. The proportion of children requiring this version of care has stayed remarkably unchanged during the existence of the team (24–27%). This speaks to the need of developing the current process to actually accommodate the integrated care needs of children with multiple neurodevelopmental problems, beyond evaluation.

Another aspect is that not all children with multiple neurodevelopmental problems are yet channelled through the Optimus integrated care model, many still having unacceptable lead time and resource use before referral. This is against the intentions of Swedish healthcare legislation, prescribing equal care to all. Thus, further implementation and scaling efforts are needed.

#### User involvement

Parents voices were integral throughout the establishment and trial of the Optimus integrated care model. Suggestions for improvement from the parent interviews above included direct access to and feedback from the special educator, information about parents’ rights when having a child with special needs, and routinely offering counselling for parents whose children have confirmed atypical development. The latter was immediately implemented, as well as information made available to resources about parents’ rights. Routine feedback from the special educator was not possible in all cases, but special educators were made aware of parents’ wishes.

Towards the end of the second year, a Family Advisory Council was established. Today, the council consists of five parents who have signed up for different capacities: supporting other parents, informing decision-makers, giving the team feedback on improvement ideas, and hosting an online group for parents. They sign up for 12 months at a time, are reimbursed for their time at an hourly rate and participate in two meetings per term with the team, apart from other activities they have signed up for. Parents from the Family Advisory Council have been instrumental in informing local politicians, decision-makers and clinic heads about the advantages of the Optimus integrated care model, generously sharing their personal stories, including previous experiences of fragmented care and its consequences for their children and families. They have also been an important resource for new parents going through the evaluation process through their mediated Facebook group where no professionals are involved.

## Discussion

In this paper we have described an integrated care strategy for pre-schoolers with suspected developmental disorders through the Optimus integrated care model. Our evaluation showed that parents were very satisfied, age at referral to Habilitation services decreased and the assessment took an average of 4.8 weeks and 5.4 clinical appointments. The Optimus integrated care model has now become standard care and is sustained, including the professionals from all the different contributing organisations.

The Optimus integrated care model is, in a sense, an example of vertical integrated care, as it includes *municipal and health* services. It is also *service integration* within the healthcare system (primary, secondary & tertiary care; the Children’s Hospital, Department of Neurology & Rehabilitation and County Habilitation services). However, there is no true vertical integration at the organisational level, and no service integration with a common payer, as defined by Ramsey and Fulop [27]. If anything, the Optimus integrated care model could be defined as *clinical integration*, although this would miss the integration of municipal special educators. The organic growth of integrated care models, such as the Optimus, thus, sometimes defies the neat theoretical conceptualisation of integrated care models. Nevertheless, the building of the Optimus integrated care model observed several of the lessons for developing integrated care, advocated by Ramsey and Fulop [27]: integrate for the right reasons; don’t necessarily start by integrating organisations; ensure local support for integration; and ensure that community services don’t miss out. These qualities might have contributed to the success and sustainability of the model.

When discussing successful integration, Jongeling et al state that “defining outcomes, utilising standardised measures, collecting systematic data, working in partnership with families to address their concerns and goals, participating in reflective practice and demonstrating a willingness to change current practice based on the results” are crucial [28]. The Optimus integrated care model has demonstrated just these capacities. Another study on successful integration of care chains found that “space for prime movers and trust between participants were crucial success factors” [29]. Thus, it seems that a number of terms regarding organisational and personnel dynamics need to be in place to initiate, develop, trial, and evaluate new models of care. As the Optimus integrated care model moves to a stage of sustainability, new challenges, not unknown in providing paediatric integrated care [19], are likely to arise.

A scoping review found that components facilitating sustainability of integrated behavioural healthcare were: “interprofessional communication and collaboration at all stages of implementation; clear protocols to facilitate intervention delivery; and co-employment of integrated care providers by specialty clinics” [19]. The Optimus integrated care model features the first two of these, whereas co-employment is not the case, although it would certainly be desirable. One of the challenges of the current model was in fact that every time leadership changed in one of the participating organisations, a new process of anchorage and buy-in had to be initiated. The primary concerns of new leaders was of economic nature, e.g., how billing should be administered, which is a common challenge in integrated care models [30]. In fact, the very benchmark of the Optimus integrated care model, the “school function clinic” at the Royal Children’s Hospital in Melbourne no longer exists, due to financial unsustainability under the current billing system that cannot accommodate multiple professionals involved in one visit. Further, a care model with several participating organisations entails a risk of diffusion of responsibility [31]. Nevertheless, the Optimus care process continues running today, without the support of the initial project leader and despite changes in staff and no one from the original development team clinically active in the team today.

In terms of balancing measures, professional time did increase in the Optimus integrated care model due to the case conference as well as the feedback session which had not been routine before. A UK study reported a median professional time of 13 hours for assessing a child with possible autism-spectrum disorder [32]: the Optimus integrated care model required less than half of this, although the difference might be partly due to the fact that no final diagnosis was made by the Optimus team. Hence, it is not uncommon for integrated care models to show some increase in cost, they are often still more cost-effective given the outcomes gained relative to the cost, compared to usual care [33]. Furthermore, if the child was not referred to another service, but still in need of intervention, this was coordinated within the team with the intervention being tailored to meet the need of the child and families with a follow-up within the team after six months. This has the potential to reduce the burden on the healthcare services, as children with developmental problems are known to be heavy service users [4,7], each child needing multiple professionals [34]. It is also worth considering that the reduction in visits would have an impact on parents’ time and work hours, known to be affected when parenting a child with a neurodevelopmental disorder [7,12].

Beyond costs, parents are asking for more integrated coordinated care where the professionals are more knowledgeable of the child and the family as well as the child’s medical history [13]. The Optimus integrated care model reduced the need for parents to recount their stories multiple times to different professionals as the anamnesis was done once with the paediatrician and the psychologist. Additionally, the case conference allowed for information to be shared as well as provided an opportunity to foster knowledge and understanding of children with neurodevelopmental disorders among the professionals. Furthermore, parents report that access to services vary greatly depending on geographical areas and professionals, as well as on parents’ own resources [10], and they are asking for an equitable distribution of support to meet the needs of the family [13]. Additionally, previously, individual health care providers felt obliged to take on a care coordinator role, which was time consuming and had the potential to lead to unequal care, delayed access to other care providers and potentially increase wait time for other families. Implementing the integrated care model may have the potential to reduce these inequalities as all children are referred to the same team and offered the same assessments by all professionals.

### User involvement in creating the Optimus integrated care model

Patient engagement is considered a cornerstone of quality of care and has been shown to improve effectiveness, efficiency, accountability, and quality of health services, as well as improve quality of life [35]. Different forms of user involvement were used throughout the project, from describing the current situation (interviews) to co-designing the integrated care model as such (patient representative present), evaluations (interviews eliciting suggestions for improvement) and finally a Family Advisory Council. In this sense, the Optimus integrated care model is an example of people-driven integrated care.

Nonetheless, there are more advanced models of co-design for paediatric care models practiced today, where large groups of parents and youth are part of the co-design process with the ambition not only to create a single care process, but reform care models, analogous [36] or digital [37]. The challenge is to create sustainable models of co-design, where users are not only present initially, but continually, building up and transforming care services, such as e.g., the learning networks for paediatric inflammatory bowel disease [38]. However, this kind of reform requires a mind shift for healthcare managers, professionals, and users alike [39].

### Limitations

Due to the observational design of this study, inferring causality or cost-effectiveness is not possible. Therefore, results should be regarded as descriptive and input to further studies using e.g., wedge-step designs when introducing Optimus-like integrated care models in clinical practice.

The approach used in this evaluation was embedded research, with its inherent pros and cons [20]. Results were continually fed back to the team, as part of the Plan-Do-Study-Act (PDSA) cycle used in quality improvement. Thus, the evaluation and development of the team became parallel and entrenched processes. Although necessary to optimise care processes according to QI methodology, this approach reduces the possibility to objectively and critically view the care process and its value. It might also reduce the ability to identify all relevant balancing outcomes. However, the evaluation of the Optimus care process extends beyond the time the project leader or the initiators of the model spent working with the team, indicating that sustainability was achieved after retraction of the embedded researchers. Nevertheless, certain limitations need to be considered. Both the project leader (A.S.) and the clinicians initially participating in the team (K.J & A.F) had a positive bias against the model and believed it would be efficient and appreciated. In addition, the embedded researchers were committed to problem solving and advocacy for the model to a greater extent than might be expected in routine clinical circumstances. Embedded research, on the other hand, provides unique insights into the problem at hand and the target group, aiding the selection of appropriate outcome measures and being able to critically appraise the extracted medical record data provided by system managers.

## Lessons learned

Although not unique to the Optimus integrated care model of developing people-driven integrated care, the lessons learned speak to the importance of goal-alignment, user involvement, attention to process and buy-in at all levels.

- Parents voices were central in creating organisational buy-in, co-designing and evaluating the Optimus integrated care model.
- Having parents in the room changed the conversation and ensured focus remained on the needs of children and their families, rather than organisational priorities and loyalties.
- Having a common body including relevant managers to anchor the project with initially, and then having the ongoing support of key managers was decisive for developing and sustaining the integrated care model, even if the structures have since shifted and several managers have moved on.
- Having a project leader initially and a designated coordinator working 20% to book appointments, lead the case conference, and liaise with managers and the Family Advisory Council were essential to the development and sustainability of the model, even if the team now has its third coordinator due to personnel turnover at the Children’s Hospital.
- Balancing measures are part of quality improvement outcomes, so also in integrated care [40]. Paying attention to, measuring, and dealing with balancing outcomes are therefore an essential part of developing and reporting integrated care models.
- Having patience with the process is truly essential [27], realising that there is a dynamic [41] where initial investment is required including managing resistance, building relationships across professional boundaries and problem solving. The payoff comes later, as does efficiency and user and team satisfaction [41].

## Conclusions

In this paper we have described the Optimus integrated care model for pre-schoolers with suspected developmental disorders. Parent satisfaction was high, the use of clinician resources efficient and the evaluation period less care than six weeks, compared to a prolonged and inefficient process before. The majority of children were referred to specialist services and at an earlier age compared to the years before the establishment of the Optimus integrated care model. The design of the evaluation warrants caution in terms of determining causality. However, the Optimus integrated care model is a relevant showcase for how people-initiated integrated care reforms can make it into usual care, under the right circumstances. This means integrating for the right reasons, leadership support, buy-in from the different organisations involved, careful process management, and the insistent involvement of users throughout the process.
